# Performance Analysis of BDS Medium-Long Baseline RTK Positioning Using an Empirical Troposphere Model

**DOI:** 10.3390/s18041199

**Published:** 2018-04-14

**Authors:** Bao Shu, Hui Liu, Longwei Xu, Chuang Qian, Xiaopeng Gong, Xiangdong An

**Affiliations:** 1 GNSS Research Center, Wuhan University, No. 129 Luoyu Road, Wuhan 430079, China; baos613@whu.edu.cn (B.S.); lw_xu@whu.edu.cn (L.X.); qc_gnss@whu.edu.cn (C.Q.); xpgong@whu.edu.cn (X.G.); xdan@whu.edu.cn (X.A.); 2 Collaborative Innovation Center of Geospatial Technology, Wuhan University, No. 129 Luoyu Road, Wuhan 430079, China

**Keywords:** BDS, zenith troposphere delay, medium-long baseline RTK, satellite geometric structure, geostationary Earth orbit satellite

## Abstract

For GPS medium-long baseline real-time kinematic (RTK) positioning, the troposphere parameter is introduced along with coordinates, and the model is ill-conditioned due to its strong correlation with the height parameter. For BeiDou Navigation Satellite System (BDS), additional difficulties occur due to its special satellite constellation. In fact, relative zenith troposphere delay (RZTD) derived from high-precision empirical zenith troposphere models can be introduced. Thus, the model strength can be improved, which is also called the RZTD-constrained RTK model. In this contribution, we first analyze the factors affecting the precision of BDS medium-long baseline RTK; thereafter, 15 baselines ranging from 38 km to 167 km in different troposphere conditions are processed to assess the performance of RZTD-constrained RTK. Results show that the troposphere parameter is difficult to distinguish from the height component, even with long time filtering for BDS-only RTK. Due to the lack of variation in geometry for the BDS geostationary Earth orbit satellite, the long convergence time of ambiguity parameters may reduce the height precision of GPS/BDS-combined RTK in the initial period. When the RZTD-constrained model was used in BDS and GPS/BDS-combined situations compared with the traditional RTK, the standard deviation of the height component for the fixed solution was reduced by 52.4% and 34.0%, respectively.

## 1. Introduction

Real-time kinematic (RTK) positioning is one of the most widely used surveying techniques given its high reliability and preferable precision. For medium-long baseline RTK, the ionosphere and the troposphere are considered two key dominant error sources that limit the capability of carrier phase ambiguity resolution (AR) and positional precision. Numerous efforts have been exerted to improve medium-long baseline RTK positioning by considering atmospheric delay [1,2,3,4]. Bock [1] proposed an ionosphere-weighted model using uncombined double-differenced (DD) observation equations, and the model was proven effective for improving the precision of float ambiguity and shortening the initialization time of RTK [3,5,6,7]. Network RTK is also an alternative approach for mitigating atmospheric errors [8,9]. In addition, a number of AR strategies have been proposed for medium-long baseline RTK [10,11,12,13,14].

However, the position error of the height component for medium-long baselines can reach a few decimeters, even after applying ambiguity resolution [15,16,17,18]. The troposphere error will be the remaining dominant factor for this effect. Even for network RTK, interpolation troposphere errors will increase when the distance between the master reference station and the user increases. The interpolated corrections for a rover also become vulnerable to localized anomalous errors under unfavorable atmospheric conditions [4,19]. For a single-reference medium-long baseline RTK, the relative zenith hydrostatic delay can be corrected in advance, and the relative zenith wet delay is usually regarded as an unknown parameter [2,10,15,19]. This method can overcome distance limitations; however, evaluated through condition numbers [18,19], researchers have pointed out that the model is ill-conditioned because the troposphere parameters are highly correlated with the height component. The regularization method can overcome the ill-conditioned problem [18], but a reasonable regularization parameter is difficult to compute. In general, the tropospheric delays are assumed as a random walk process, and thus a filtering technique is used [20,21]. The filter enables us to distinguish the troposphere zenith delay from the height component with changes in satellite geometry and mapping functions [21]. However, a relatively long period is required to initialize the troposphere parameter, and although it has been alleviated, the ill-conditioned problem remains. 

Additional difficulties will occur in terms of the troposphere and height solution when BeiDou Navigation Satellite System (BDS) observations are used in medium-long baseline RTK. BDS can provide reliable positioning results by itself [22,23,24]. Together with GPS, the ambiguity fixed rate and the precision of single-epoch RTK are increased [14,25,26]. However, BDS has three different orbit types: geostationary Earth orbit (GEO), inclined geosynchronous orbit (IGSO), and medium Earth orbit (MEO); therefore, its observation time, geometric structure, and change rate of satellite geometry differ from those of the GPS system. When considering the strict relationship between geometric structure and the troposphere parameter, different characteristics will be observed in BDS medium-long baseline RTK.

Meanwhile, troposphere corrections derived from global zenith troposphere delay (ZTD) models now have good precision. The accuracy of the widely-used troposphere-corrected model UNB3 can reach 5.2 cm, whereas that of the modified model UNB3 m is approximately 4.8 cm [27]. The first global pressure and temperature (GPT) model was also proposed based on the ERA-40 of the European Center for Medium-Range Weather Forecasts [28]. Subsequently, improved versions such as GPT2 [29] were developed. Yao [30] proposed a ZTD-constrained precise point positioning (PPP) model using virtual ZTD observations to accelerate PPP convergence. The effect of troposphere-enhanced PPP has been validated by a number of scholars [31,32,33]. Along with accelerating convergence time, the results also show that the new method can improve the accuracy of the PPP solution. Accounting for the serious ill-conditioned problem in medium-long baseline RTK and the special satellite structure for the BDS system, introducing external troposphere information for BDS medium-long baseline RTK is expected to be valuable, and merits an investigation.

Therefore, the main purpose of this study is to assess the performance of BDS medium-long baseline RTK using a priori empirical troposphere information. First, the principle of the RZTD-constrained medium-long baseline RTK model is introduced. Then, the factors affecting the precision of BDS RTK positioning are analyzed and compared with GPS performance. Finally, the effects of the RZTD-constrained GPS, BDS, and GPS/BDS-combined RTK are evaluated using 15 baselines ranging from 38 km to 167 km.

## 2. Methodology

In this section, we first present the traditional DD observation model for medium-long baseline RTK positioning, and then the RZTD-constrained model is introduced in detail.

### 2.1. Traditional Observation Model for Medium-Long Baseline RTK

The uncombined DD observation model is adopted for medium-long baseline RTK in this study. The basic DD pseudo-range and carrier phase equations are as follows:

(1) $$P_{rb,k}^{ij}=\rho_{rb}^{ij}+\mu_{k}I_{rb}^{ij}+(\alpha_{r}^{j}-\alpha_{r}^{i})T_{r}-(\alpha_{b}^{j}-\alpha_{b}^{i})T_{b}+\varepsilon_{P,k},$$

(2) $$L_{rb,k}^{ij}=\rho_{rb}^{ij}-\mu_{k}I_{rb}^{ij}+(\alpha_{r}^{j}-\alpha_{r}^{i})T_{r}-(\alpha_{b}^{j}-\alpha_{b}^{i})T_{b}+\lambda_{k}N_{rb,k}^{ij}+\varepsilon_{L,k},$$

where L and P represent the DD pseudo-range and carrier phase observations in units of length, respectively; ρ is the geometric range between the satellite and the receiver antenna phase center; T denotes the zenith troposphere delay that can be converted into the slant delay with the mapping coefficient α; λ is the wavelength; k is the signal frequency number; N is the integer ambiguity; μ is the coefficient that can convert the ionosphere delay on frequency 1 into frequency k with $\mu_{k}=f_{1}^{2}/f_{k}^{2}$; $\varepsilon_{P,k}$ and $\varepsilon_{L,k}$ represent the observation noise of the pseudo-range and the carrier phase, respectively; r and b denote the rover and base station, respectively; j is the non-reference satellite; and i is the reference satellite.

For medium-long baseline, the DD residual wet tropospheric delay can be approximately represented by a function of relative ZTD (RZTD) and a mapping function [2,34]. This means that the troposphere parameters $T_{r}$ and $T_{b}$ in (1) and (2) can be combined into a single parameter as follows:

(3) $$(\alpha_{r}^{j}-\alpha_{r}^{i})T_{r}-(\alpha_{b}^{j}-\alpha_{b}^{i})T_{b}≅\alpha^{ij}\cdot T_{rztd},$$

where $\alpha^{ij}$ is the coefficient of RZTD $T_{rztd}$ and $\alpha^{ij}=\alpha^{j}-\alpha^{i}$, the mapping coefficient α is the function of satellite elevation angle, and the elevation angle parameters for $\alpha^{i}$ and $\alpha^{j}$ are the average elevation angles of two receivers for satellites i and j, respectively [34]. The global mapping function (GMF) [35] is used in this study.

The RTK observation equations—in which the baseline components, the ambiguities, and the slant ionosphere and zenith troposphere delays are unknown parameters—are given as follows:

(4) $$E\left[\begin{array}{c} P\\ L \end{array}\right]=\left[\begin{array}{cccc} A & \Lambda & B & 0\\ A & -\Lambda & B & C \end{array}\right]\left[\begin{array}{c} a\\ b\\ c\\ d \end{array}\right],$$

where E[⋅] denotes the expectation operator; P and L denote the DD pseudo-range and the carrier phase vector, respectively; a is the baseline vector and the corresponding coefficient matrix A contains the line-of-sight unit vectors between the two receivers; b denotes the DD ionosphere delay vector and the corresponding matrix Λ contains the coefficient $\mu_{k}$ for all frequencies; c is the RZTD parameter and the corresponding vector B contains mapping coefficients between two receivers; and d is the DD ambiguity vector and the corresponding coefficient matrix C contains the wavelength vectors for carrier phase observations. For multi-constellation signals, we apply system-specific DD and thus have one pivot satellite per system [14]; the baseline vector and RZTD parameters are common, and the ionosphere and ambiguity parameters are specific to the respective system.

### 2.2. RZTD-Constrained RTK Model

The troposphere parameters are highly correlated with the height component, particularly for satellites with high elevation angles [19,20]. In an ill-conditioned model, a small error in the observation will cause a large error in the result. For GPS medium-long baseline RTK, the position error of the height component can reach a few decimeters, even if the ambiguities are correctly fixed [15,17,18]. Compared to the GPS system, the height component for the BDS system may be affected considerably by the introduction of the troposphere parameters because of the heterogeneous satellite distribution. However, if the troposphere parameters are properly constrained by precise a priori RZTD information, the ill-conditioned problem can be alleviated and a better position solution can be obtained for BDS. In the RZTD-constrained model, the precise a priori troposphere correction $T_{rztd}^{0}$ and the corresponding variance should be provided as follows:

(5) $$T_{rztd}^{0}=T_{rztd}+\varepsilon_{T_{rztd}^{0}}\;\sigma_{T_{rztd}^{0}}^{2}.$$

In the current study, a priori zenith wet troposphere delays are derived from the high-precision GPT2 model [29], and $\varepsilon_{T_{rztd}^{0}}$ is the error of the a priori troposphere information. $\sigma_{T_{rztd}^{0}}^{2}$ is the variance. A smaller troposphere variance means a tighter constraint, thus the height component can be more easily distinguished from the troposphere parameter. These troposphere corrections are introduced as pseudo-observations together with the pseudo-range and carrier observations into every epoch. Meanwhile, we regard the RZTD parameter as a random walk process. The corresponding state transition equation between two consecutive epochs is:

(6) $$T_{rztd,m}=T_{rztd,m-1}+w_{rztd,m}\;\mathrm{with}\;w\sim N(0,\sigma_{w}^{2}),$$

where w is the process noise, which obeys Gaussian distribution $N(0,\sigma_{w}^{2})$, $\sigma_{w}^{2}=q_{w}\cdot \Delta t$, Δt is the time interval between two consecutive epochs, and $q_{w}$ is the spectral density. Combining Equations (4) and (5), the unknown parameters can be estimated by extended Kalman filter.

## 3. Experiment and Result Analysis

In this section, first the experimental data and processing strategy are introduced, then factors affecting the precision of the BDS medium-long baseline RTK and the performance of the RZTD-constrained RTK model are analyzed.

### 3.1. Data Collection and Processing Strategy

**Figure 1:**
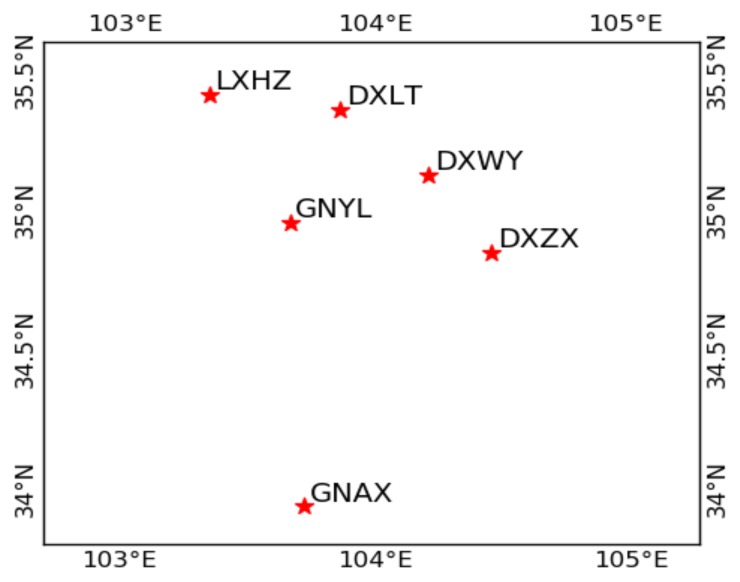
Distribution of the six continuously operating reference stations (CORSs).

The experimental data are from six continuously operating reference stations (CORSs) at the medium latitude of China. The distribution of the six stations is shown in Figure 1. A total of 15 medium-long baselines ranging from 38 km to 167 km are formed by these six stations. All the stations are equipped with Trimble NetR9 receivers and TRM59900.00 antennas. The length and elevation differences of all the baselines are listed in Table 1. Four days of real dual-frequency GPS/BDS data were collected in 2017, the interval of the data was 30 s, and the cutoff elevation angle was set as $10^{{}^{\circ}}$. The GPS, BDS, and GPS/BDS-combined data from each baseline were carefully processed using the traditional and RZTD-constrained RTK models. Considering the possible wrongly fixed ambiguities in past epochs, instead of inheriting the fixed ambiguity, the ambiguity of current epochs will try to fix them again. The processing strategies of traditional and RZTD-constrained RTK are illustrated in Table 2. The empirical initial values of ambiguity, ionosphere, and RZTD parameters together with their corresponding variances in the Kalman filter were set in the same way for the two models. The process noise of the RZTD parameter was set to 0.1 mm/$\sqrt{s}$ [10] for the two models. Different from the traditional model, the external troposphere corrections derived from the GPT2 model were introduced as pseudo-observations into every epoch for the RZTD-constrained RTK model. Together with the data from a winter day (DOY016), data from three days in the other three seasons (DOY092, 190, 289) were processed to compare the effect of the RZTD-constrained RTK model in different troposphere conditions. 

The ambiguity fixed rate is one indicator to assess the performance of RTK positioning. For medium-long baseline RTK, the multipath error and the residual atmospheric delays will reduce the success rate of AR. In this study, the partial ambiguity fixing (PAR) technique is proposed when a full set of ambiguities cannot be fixed. The first step is to form wide-lane (WL) and narrow-lane (NL) ambiguities from the original ambiguity (L1/L2 or B1/B2 ambiguity, derived from Section 2.2) using a transformation matrix [13]; the corresponding variance–covariance matrix for WL and NL ambiguities are also computed by the law of error propagation. The LAMBDA method [36] is applied to search for the optimal fixed value of the ambiguities; after WL ambiguities are fixed, the NL ambiguities begin to fix. In order to obtain a reliable ambiguity-fixed solution, the fixed-failure rate ratio test (FF-ratio) test [37] is applied for ambiguity validation; the fixed-failure rate is set as 1%. If the ambiguity validation fails, the ambiguity subset is selected by excluding the ambiguities whose best candidates differ from second-best candidates [12], and the selected ambiguity subset will be fixed with the LAMBDA method again. To ensure the accuracy of the fixed solution, if they pass the FF-ratio test and the number of fixed NL ambiguities is not fewer than 6, these integer ambiguities will be applied as constraints to update coordinate parameters. The fixed rate of AR is defined as follows:

(7) $$P_{fixed}=\frac{N_{\mathrm{fixed}}}{N_{total}}\times 100\%,$$

where $N_{\mathrm{fixed}}$ refers to the epoch number of the fixed solution (fixed NL ambiguities are no fewer than six) and $N_{total}$ represents the total number of epochs.

Time to first fix (TTFF) is the time to initially fix ambiguity, which is another indicator to evaluate the performance of RTK positioning. To calculate the average TTFF, the ambiguities and atmospheric parameters were reset repeatedly for all baselines during the experiment:

(8) $$Average\;TTFF=\frac{\sum_{i=1}^{n}TTFF_{i}}{n},$$

where n is the number of sessions. In this study, the time span was set as 4 h for each session in order to acquire enough samples for calculating TTFF; data from 0:00:00–3:59:30, 0:30:00–4:29:30, 1:00:00–4:59:30,……, 20:00:00–23:59:30 were processed; and a total of 41 sessions were obtained for each baseline in one day—six of them independent sessions (0:00:00–3:59:30, 4:00:00–7:59:30,……, 20:00:00–23:59:30).

### 3.2. Performance Analysis of Traditional Medium-Long Baseline RTK

Prior to evaluating the RZTD-constrained model, GPS, BDS, and GPS/BDS-combined data were processed with the traditional RTK model. Thus, the factors affecting the precision of BDS medium-long baseline RTK positioning were analyzed and compared with the GPS performance.

**Figure 2:**
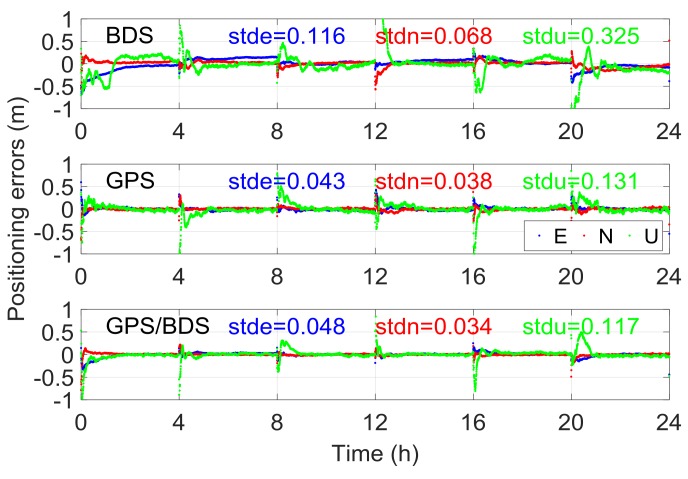
Float positioning results using the traditional real-time kinematic (RTK) model for Baseline 6.

**Figure 3:**
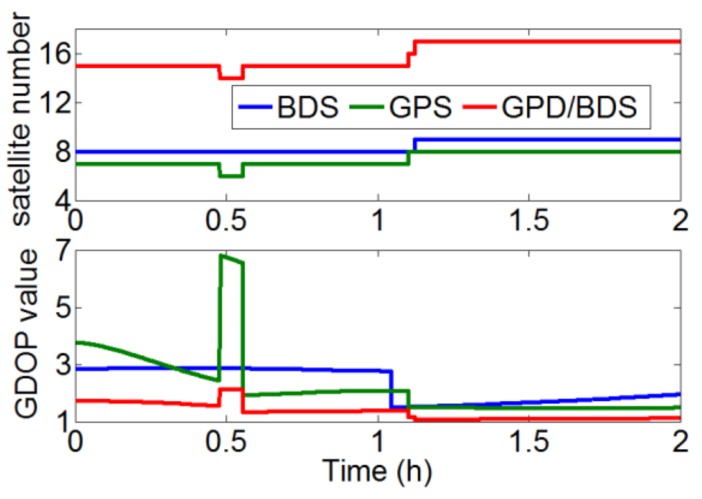
(**top**) Satellite numbers and (**bottom**) geometric dilution of precision (GDOP) values of BDS, GPS, and GPS/BDS-combined situations.

**Figure 4:**
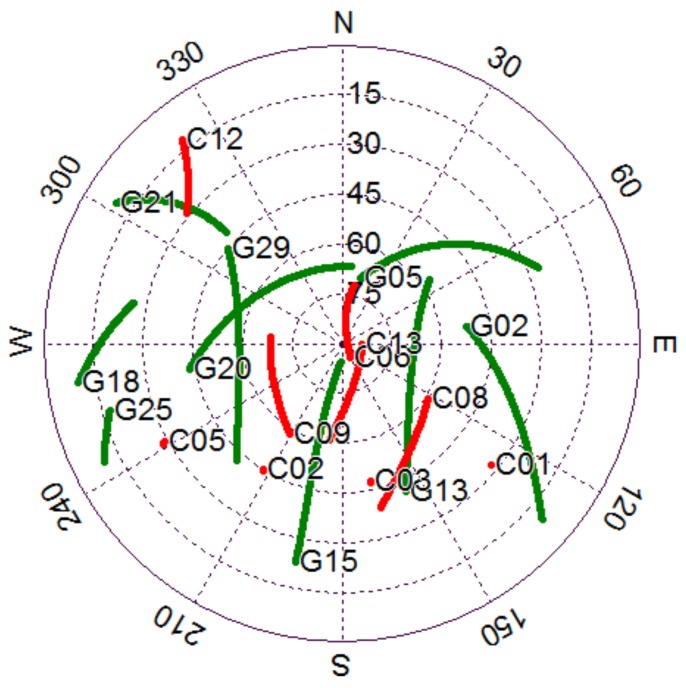
Sky plots (azimuth vs. elevation) for the rover station of Baseline 6 from 00:00:00 to 02:00:00 (red: BDS, green: GPS).

**Figure 5:**
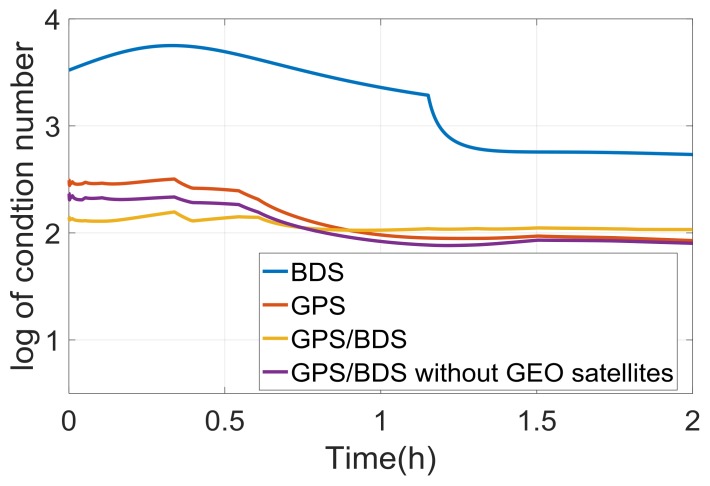
Condition numbers of the four situations in the Kalman filter process. GEO: geostationary Earth orbit.

A precise float ambiguity is a prerequisite for a reliable AR. To a certain extent, the float RTK positioning solution can reflect the precision of float ambiguity and other correlated parameters. Figure 2 illustrates the traditional float RTK positioning results from six independent sessions on DOY016 solved with BDS, GPS, and GPS/BDS data. Due to space limitations, only the result of one baseline (here Baseline 6) is presented. As shown in the figure, the standard deviations (STDs) of the three baseline components for BDS RTK positioning were 0.116, 0.068, and 0.325 m, which are apparently higher than those for GPS (0.043, 0.038, and 0.131 m) and GPS/BDS (0.048, 0.034, and 0.117 m), particularly for the height direction. The height error of BDS for traditional RTK was over 0.5 m, even after 1 h of initialization in the first session. Figure 3 shows the satellite number and geometric dilution of precision (GDOP) values of the three situations for the first 2 h. Notably, the number of satellites for BDS is one or two more than that for GPS. However, the GDOP values of GPS are lower than those of BDS in most cases. As shown in Figure 4, the weak satellite geometric structure of BDS may be responsible for this result. The BDS satellites are more unevenly distributed than the GPS satellites. These two items are indicators of satellite geometric structure in a single epoch. The filter estimator captures the changes in satellite geometry and mapping functions over a certain period. This capability of the filter estimator enables us to distinguish the troposphere zenith delay from the height component. The condition numbers of BDS, GPS, and GPS/BDS scenarios were computed in the Kalman filter process. The condition number is defined by the ratio of maximal and minimal eigenvalues of the normal matrix for the estimates. This is a measure of how numerically well-conditioned or ill-conditioned a problem is [18,19]. As shown in Figure 5, with changes in satellite geometry, the condition number decreased over time and tended to become stable after a certain period. Simultaneously, the condition number of BDS was significantly higher than those of GPS and GPS/BDS-combined. The condition number of BDS was over $10^{2.5}$, even after convergence, which is higher than the value of GPS for the first epoch. A high condition number means a serious ill-conditioned problem, and thus the height component is difficult to distinguish from the troposphere parameter. Consequently, the low precision of the height component for BDS appears reasonable due to the poor satellite geometric distribution and the ill-conditioned problem. In addition, it can be seen that the GPS condition numbers went below the GPS/BDS model in Figure 5 after a longer time-span filtering. Compared with condition number of GPS/BDS without GEO satellites, we can conclude that this is mainly attributable to the BDS GEO satellites. With GEO satellites, the ill-condition number can be alleviated in the initial period; however, the geometry of GEO satellites remains almost unchanged—they may even hinder the reduction of condition number as time goes by.

**Figure 6:**
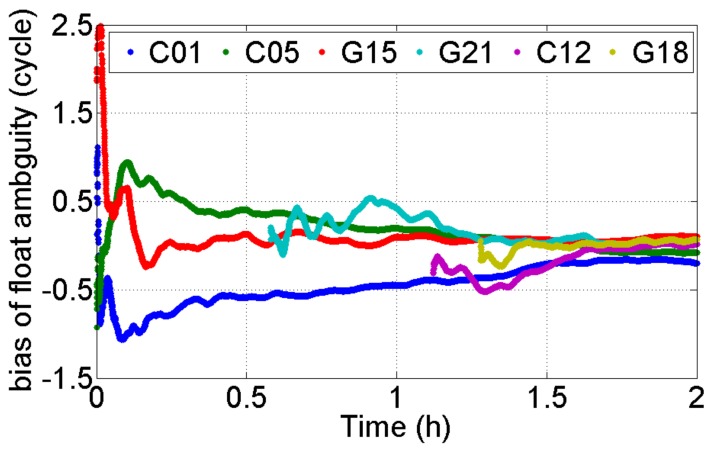
Bias of float ambiguities for satellites with a low elevation angle from 00:00:00 to 02:00:00.

In principle, the geometric structure of GPS/BDS is considerably better than that of single GPS. However, a confused phenomenon is shown in Figure 2. That is, the precision of the height component for the GPS/BDS-combined situation was lower than that for the single GPS system in the initial period (e.g., sessions 1 and 6). In fact, the precision of float RTK positions will also be influenced by the precision of float ambiguities, particularly for satellites with a low elevation angle. Figure 6 shows the bias of float ambiguities for satellites with low elevation angle when the aforementioned baseline was solved using GPS/BDS data during this period. The biases of float solutions were determined by subtracting integer ambiguities from float solutions. The initialization time of BDS satellites C01 and C05 was considerably longer than that of the other satellites. Notably, C01 and C05 are GEO satellites with positions that remain nearly static with respect to ground stations. GEO satellites lack variation in geometric structure, hence ambiguity convergence time is considerably longer than that of satellites with other orbit types when the elevation angle is relatively close. Although the GEO satellites have some disadvantages regarding parameter initialization, the positioning performance will be improved after their ambiguities convergence. 

### 3.3. Performance Analysis of RZTD-Constrained Medium-Long Baseline RTK

To alleviate the ill-conditioned problem and improve height precision in medium-long baseline RTK, the GPT2-constrained RTK model was used. The performance of RTK positioning were assessed based on this empirical model, and results are presented in this section. For fixed solutions, the TTFF, ambiguity fixed rate, and precision of the fixed solution were analyzed compared with the traditional RTK. In addition, the effect of the RZTD-constrained RTK model were analyzed with the data in different troposphere conditions.

**Figure 7:**
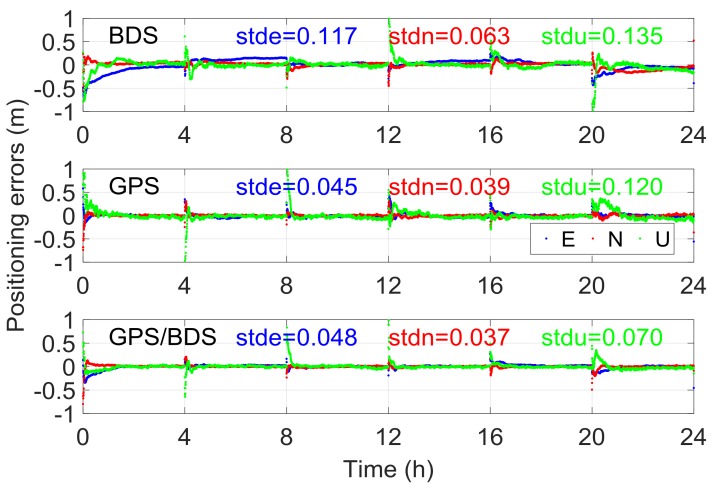
Float RTK positioning result using GPT2-constrained RTK model for Baseline 6.

GPT2 is a global empirical model that provides troposphere delays. First, pressure, temperature and its lapse rate, water vapor pressure, and the hydrostatic and wet mapping function coefficients were determined for specific sites near the surface of the Earth. Thereafter, considering these meteorological parameters, the zenith hydrostatic delay (ZHD) and zenith wet delay (ZWD) were calculated using the equation in the Saastamoinen model [38]. Compared with ZTD provided by 341 IGS stations for one year, the mean bias and root mean square (RMS) errors of the GPT2 model were about 0.28 and 3.79 cm [39]. As a result, the σ for the a priori RZTD information in Equation (5) for every epoch was temporarily set as 4 cm here; the effects of different variances will also be discussed in next section. Figure 7 presents the float RTK positioning results of six independent sessions for the aforementioned baseline, where the GPT2-constrained RTK model was implemented. The STDs of the height component for BDS, GPS, and GPS/BDS were 0.135, 0.120, and 0.070 m, respectively, which were improved by 58.5%, 8.4%, and 40.2%, respectively, compared with the traditional RTK (Figure 2). Evidently, significant reductions in height deviation occurred in the initial period. The large fluctuations for the traditional BDS RTK shown in Figure 2 also disappeared. In terms of the eastern and northern components, nearly no improvement occurred compared with Figure 2. Although the positioning results from the two methods became comparable after convergence, the GPT2-constrained RTK solution tended to be more stable and exhibit fewer variations. 

**Figure 8:**
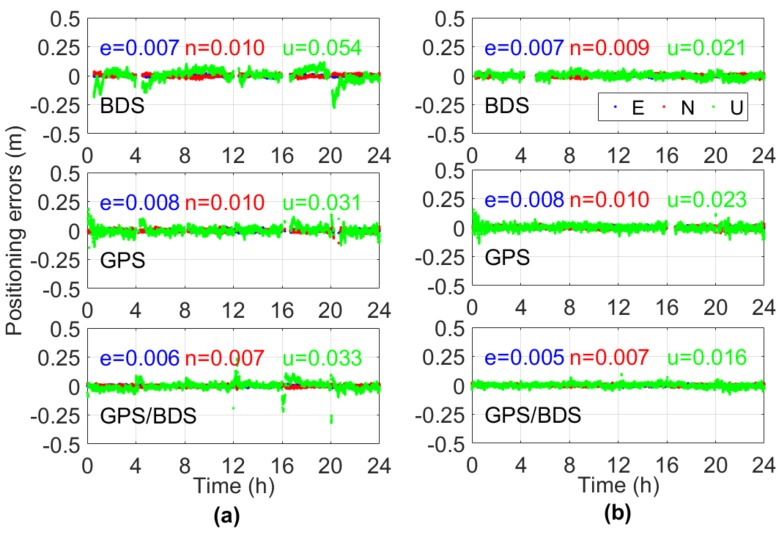
Fixed RTK positioning results solved using the (**a**) traditional and (**b**) GPT2-constrained models. The letters e/n/u represent the standard deviation (STD) of position results in the east/north/up directions, respectively.

The RTK fixed solution was determined for all 15 baselines on DOY016 given the AR strategies in Section 3.1. The ambiguity-fixed east/north/up coordinate solutions using the traditional and GPT2-constrained RTK model for six independent sessions of Baseline 6 are shown in Figure 8. The fixing rate of BDS was 82.7% for traditional RTK and 87.9% for GPT2-constrained RTK, an improvement of 6.3%. The fixing rate of GPS/BDS was 98.9% for the GPT2-constrained RTK, which was nearly unchanged compared with 97.9% for the traditional RTK. A possible reason for this is that the geometry of GPS/BDS is considerably better than that of single BDS. The contribution of external troposphere corrections to float ambiguity was not as apparent for GPS/BDS compared with single BDS. In terms of the accuracy of the fixed solution, the eastern/northern component was nearly unchanged, similar to that in the float solution. However, the height accuracy of the GPT2-constrained model improved considerably compared with the traditional RTK. The height STD of the GPT2-constrained RTK model was 0.021 m for BDS, which was improved by 61.1% over the 0.054 m in the traditional model. In addition, for the BDS of traditional RTK, the height error was over 0.1 m for a certain percentage of epochs, whereas the height error for all epochs was less than 0.1 m for GPT2-constrained RTK. In the GPS/BDS situation, the accuracy of the height component was improved by 51.5% and the abnormal values in the traditional RTK disappeared in the GPT2-constrained RTK. The improvement of the height component was 25.8% for GPS, which was less than that for BDS and GPS/BDS. 

**Figure 9:**
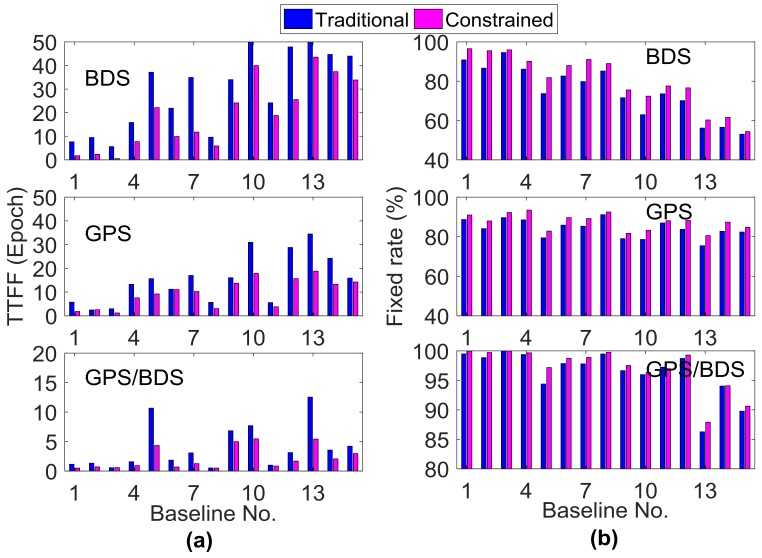
(**a**) Time to first fix (TTFF) and (**b**) fixed rate of the fixed solution using traditional and GPT2-constrained RTK.

**Figure 10:**
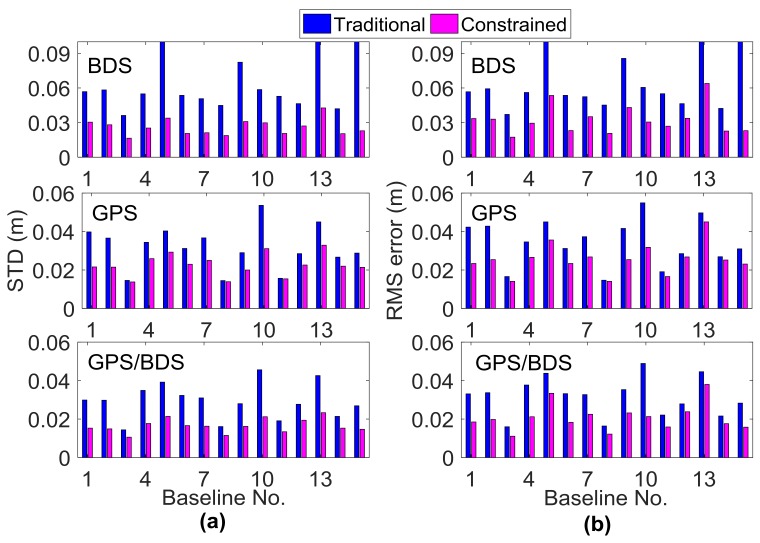
(**a**) Vertical STD and (**b**) root mean square (RMS) error of the fixed solution using traditional and GPT2-constrained RTK.

As shown in Figure 8, the effect of the external troposphere correction on the horizontal accuracy of RTK positioning was not apparent; thus, only the height component is shown in the following analysis. Figure 9 and Figure 10 illustrate the statistical results of TTFF, fixed rate, STD, and RMS errors in the vertical direction for all 15 baselines on DOY016. As introduced in Section 3.1, for each baseline, the TTFF was calculated using the results of 41 sessions; the remaining three indicators were calculated using six independent sessions, in order to avoid duplication of statistics. The reference coordinates were determined by the post-processing GAMIT software [40]. It can be seen that TTFF was accelerated when the GPT2-constrained model was used. Generally, BDS showed longer TTFF, and the TTFF of two baselines was even more than 50 epochs (30 s interval) for the traditional model; this may be due to the heterogeneous satellite distribution and long convergence time of the ambiguity parameter for GEO satellites. Compared to GPS and BDS, GPS/BDS showed great improvement in TTFF; the TTFF of 11 baselines was less than three epochs in the GPT2-constrainted model. In terms of ambiguity fixed rate, all 15 baselines improved using the GPT2-constrained model. For BDS, Baselines 7, 10, and 12 exhibited an improvement of 14.1%, 15.0%, and 9.3%, respectively; the average improvement was approximately 7.5%. However, only a slight improvement was observed for the GPS/BDS-constrained RTK model. Meanwhile, the fixing rate decreased as the baseline length became longer. A large residual atmospheric error—particularly for ionosphere delay—may account for such a result. In addition, the STD of the height component was reduced considerably. The height STDs of 11 baselines were reduced by over 50% for BDS, whereas the STDs of 10 baselines were reduced by over 40% for GPS/BDS. The STDs of the fixed results for three exceptional baselines were over 0.1 m. Incorrectly-fixed ambiguities for certain epochs were responsible for this situation. The correct integer ambiguities were judged by comparing the ambiguities derived from RTK mode with post-processing mode. The lengths of the two baselines were 138.9 km and 166.9 km, respectively. A long baseline will have a high probability of being incorrectly fixed, particularly for weak geometric structure conditions such as the BDS-only situation. In addition, the STD of Baseline 5 for BDS traditional RTK was also more than 0.1 m, which was similarly caused by incorrectly-fixed ambiguities for certain epochs. However, the STD became normal when the GPT2-constrained RTK model was used, indicating that external troposphere corrections were helpful in improving model strength and float ambiguity accuracy. The RMS error of the positioning results for the GPT2-constrained RTK model also decreased considerably compared with that for the traditional RTK model. The average performance improvement of height RMS error was 44.2% and 33.1% for BDS and GPS/BDS situations, respectively. 

**Figure 11:**
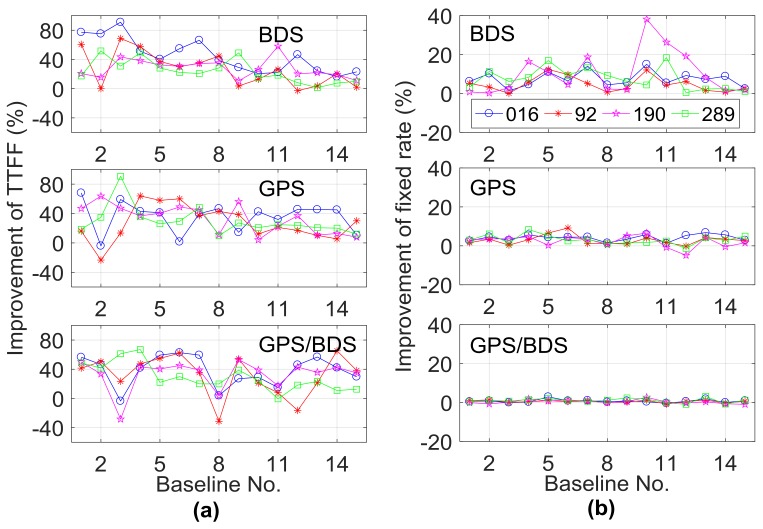
Improvement of (**a**) TTFF and (**b**) fixed rate on DOY016, 92, 190, and 289 when comparing the GPT2-constrained model with the traditional model.

**Figure 12:**
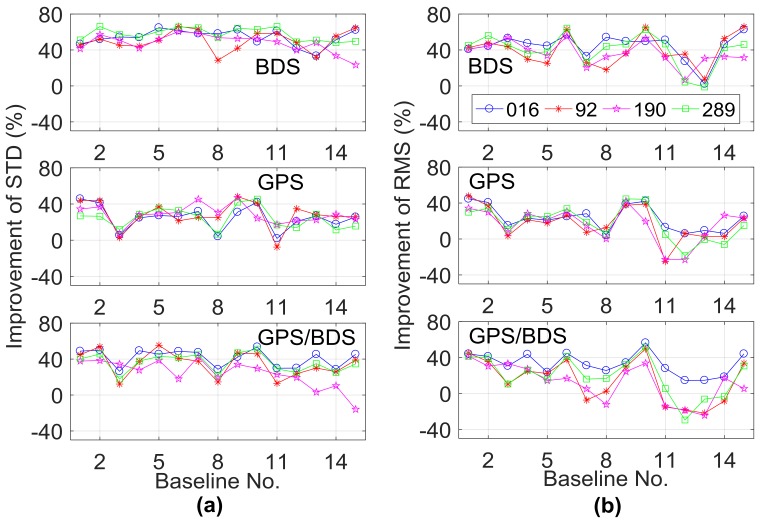
Improvement of (**a**) STD and (**b**) RMS on DOY016, 92, 190, and 289 when comparing the GPT2-constrained model with the traditional model.

To analyze the effect of the GPT2-constrainted RTK model in different troposphere conditions, the data from the other three days (DOY092, 190, 289) in spring, summer, and autumn were processed, and the σ for the a priori RZTD in Equation (5) was set as 4 cm. Figure 11 and Figure 12 illustrate the performance improvements in terms of TTFF, fixed rate, vertical STD, and RMS when comparing the GPT2-constrained model with the traditional RTK model. Table 3 shows the average statistical results of 15 baselines for the four indicators. The improvement is calculated as follows:

(9) $$Improvement\;rate=\alpha \frac{v_{c}-v_{t}}{v_{t}}\times 100\%,$$

where $v_{t}$ and $v_{c}$ are the values of these four indicators for the traditional model and constrained model and α is the coefficient, with α equal to 1 for the ambiguity fixed rate indicator and –1 for the remaining three indicators.

In terms of TTFF, comparing the results on DOY016, the performance of BDS decreased on the other three days, whereas the GPS and GPS/BDS-combined scenarios did not show apparent performance reduction. The performance of the fixed rate indicator showed a slight difference for the four days in different seasons. Compared to the results on DOY016, the performance of STD and RMS indicators on the other three days showed different levels of reduction for the GPS/BDS-combined scenario. Specifically, the performance reduction on DOY190 was most obvious. On DOY016, the average improvement of STD and RMS indicators for the 15 baselines was 41.3% and 33.1% for the GPS/BDS-combined scenario, while the corresponding values were 24.0% and 12.1% on DOY190. The atmospheric circumstance varies greatly in the summer, the precision of the empirical GPT2 model becomes uncertain, and the performance of the RZTD constrained model may also be reduced. Compared to the GPS/BDS-combined situation, only slight performance reduction was observed in the BDS-only scenario for these two indicators on DOY190. The satellite geometry structure of the BDS-only scenario was weak; with the external constraint, the performance could be greatly improved even in relatively tricky tropospheric conditions. Meanwhile, compared to STD, the performance of the RMS indicator showed a larger reduction; for example, on DOY190, the improvement of the STD indicator for Baselines 11–13 was positive for the GPS/BDS-combined situation, whereas the improvement of the RMS indicator became negative for these three baselines. It should be noted that the length of these three baselines was more than 100 km. The a priori RZTD corrections have larger uncertainty when the baseline is longer and the external troposphere constraint may result in position bias. In this case, the variance of a priori RZTD information also matters, which will be discussed in the next section. In general, the GPT2-constrained model can improve the performance of medium-long RTK in different tropospheric conditions. For BDS, the average performance improvement of TTFF, fixed rate, STD, and RMS for the four days were 30.9%, 7.5%, 52.4%, and 40.0%, respectively. The corresponding values for the GPS/BDS-combined scenario were 33.0%, 0.7%, 34.0%, and 19.8%.

## 4. Discussion

The external constraint can help alleviate the ill-conditioned problem and improve the accuracy of estimated parameters in medium-long baseline RTK; however, the external constraint may also result in a bias for the other parameters when the corresponding variance $\sigma^{2}$ is too small. In contrast, if $\sigma^{2}$ is excessively large, then the contribution of constraints will fade. Possible introduced bias and the positive effect on height precision of external tropospheric corrections should be balanced. The improvement rates of STD indicators on a summer day were lower than on a winter day based on the analysis in the previous section (Table 3). Negative values even appeared for the improvement rates of some baselines. This indicates that position bias may have been introduced because of external constraints. In addition, the precision of the a priori tropospheric corrections derived from the GPT2 model may also vary at different times and areas. Consequently, an appropriate σ should be set for external constraints.

**Figure 13:**
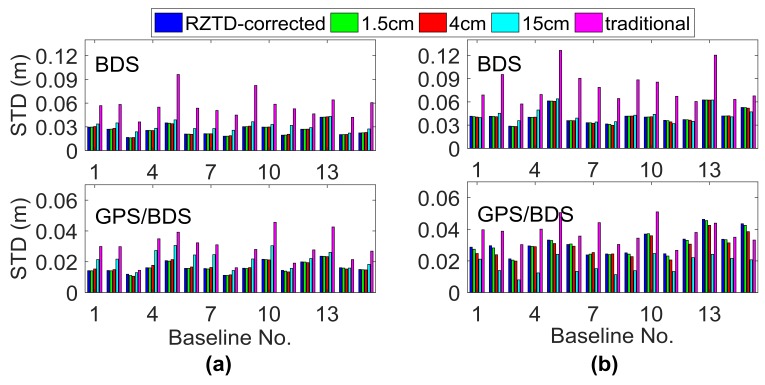
STD of the fixed RTK solution on (**a**) DOY016 and (**b**) DOY190 with different STD σ for the a priori GPT2 tropospheric corrections.

The data collected on DOY016 and 190 for all 15 baselines were processed using the GPT2-constrained RTK model with different STD σ for the a priori GPT2 tropospheric corrections. Figure 13 shows the vertical STDs of the fixed RTK solution for BDS-only and GPS/BDS-combined scenarios when σ for external RZTD information in Equation (5) was set as 1.5, 4.0, and 15 cm, respectively. The statistical results only included the correctly fixed solution. The traditional RTK model can be regarded as the RZTD-constrained RTK model when σ is set as ∞ for GPT2 troposphere information. In addition, without estimating the RZTD parameter, the method correcting the troposphere error in phase and pseudo-range observations with the GPT2 model was also used for comparison. This method is also called the ZTD-corrected model, which can be regarded as the RZTD-constrained model when σ is set infinitely close to zero. Compared to the BDS-only situation, GPS/BDS-combined RTK seems to be more sensitive to the value of σ. In two different tropospheric conditions, compared to traditional RTK, the STDs of the BDS-constrained RTK solution greatly decreased when σ was set between 1.5 cm and 15 cm; however, the performance of the GPS/BDS-combined GPT2-constrained RTK varied greatly with different σ. Additionally, it can be seen that on DOY016, for most baselines, the STD of the positioning results increased for both BDS-only and GPS/BDS-combined scenarios when the value of σ increased, and the RZTD-corrected model obtained a preferable effect on this day. However, on DOY190, the STD of the BDS-only fixed solution for Baselines 11, 12, 14, and 15 did not increase as the value of σ increased; the STD of the GPS/BDS-combined RTK for most baselines greatly decreased when the value of σ varied from 1.5 cm to 15 cm, and the performance of the RZTD-corrected model was the worst for the GPS/BDS-combined situation on this day. This means that for relatively stable tropospheric conditions on a winter day, a smaller σ value seems preferable. However, on a summer day, a higher σ value is helpful to decrease possible introduced bias. In general, to ensure the reliability of the RZTD constrained model, a relatively larger variance is usually proposed.

## 5. Summary and Conclusions

This study was aimed mainly at the ill-conditioned problem in medium-long baseline RTK. The height component is highly correlated with troposphere parameters and was unstable in the medium-long baseline RTK solution. For the BDS system, due to a lack of variation for BDS GEO satellites and nonhomogeneous satellite distribution, the height component was more difficult to distinguish from the troposphere zenith delay compared with the GPS system. As a result, the performance of BDS medium-long baseline RTK was worse than that of GPS, particularly for the height direction. Even for the GPS/BDS-combined RTK, although the geometric structure is evidently improved, due to the lack of variation in geometry structure, the convergence time of ambiguities for the BDS GEO satellite was significantly longer than that of satellites with other orbit types, which may result in lower precision in height direction in the initial period.

To reduce the ill-conditioned problem and alleviate the negative effects of BDS GEO satellites, the GPT2-constrained RTK model was used and evaluated. In the case of 15 baselines at the medium latitude of China, with a variance of 4 × 4 cm^2^, the results show that the ambiguity fixed rate of BDS was improved by approximately 7.5%, whereas only a slight improvement was observed for GPS/BDS when the GPT2-constrained RTK model was used. Compared with the traditional RTK model, the STD of the height component was reduced by an average of 52.4% and 34.0% for BDS-only and GPS/BDS-combined situations, respectively. In terms of RMS error, the average performance was improved by 40.0% and 19.8% for the BDS and GPS/BDS-combined situations, respectively. It should be noted that this method could improve the performance of medium-length RTK only to a certain degree. For the bad satellite geometric structure, it is also difficult to acquire satisfying results. Meanwhile, the accurate a priori tropospheric information is difficult to obtain in some conditions (e.g., long baselines, large elevation differences, or bad weather conditions). As a result, this method is applied to baselines with medium length. In addition, a larger variance is usually proposed to ensure reliability. 

## Figures and Tables

**Table 1:** Baseline information.

Baseline	No.	Length (km)	Elevation Difference (m)	Baseline	No.	Length (km)	Elevation Difference (m)
DXWY-DXZX	1	38.3	214	DXWY-LXHZ	9	85.5	66
DXWY-DXLT	2	41.2	160	GNAX-GNYL	10	112.9	311
DXLT-LXHZ	3	47.7	271	DXZX-LXHZ	11	119.8	280
GNYL-DXLT	4	48.3	310	GNAX-DXZX	12	121.4	8
GNYL-DXWY	5	53.6	105	GNAX-DXWY	13	138.9	206
GNYL-LXHZ	6	58.4	39	GNAX-DXLT	14	158.0	1
GNYL-DXZX	7	74.0	319	GNAX-LXHZ	15	166.9	272
DXZX-DXLT	8	78.9	9	-		-	-

**Table 2:** Processing strategies of traditional and relative zenith troposphere delay (RZTD)-constrained real-time kinematic (RTK) model. DD: double-differenced; GMF: global mapping function; GPT: global pressure and temperature; *BL*: baseline length in kilometers.

	Traditional RTK	RZTD-constrained RTK
Observations (variance)	Uncombined DD pseudorange ($\sigma_{P}^{2}$m)	Uncombined DD pseudorange ($\sigma_{P}^{2}$m)
	Uncombined DD carrier phase ($\sigma_{L}^{2}$m)	Uncombined DD carrier phase ($\sigma_{P}^{2}$m)
Pseudo-observations (variance)	-	RZTD derived from GPT2 model ($\sigma_{T_{rztd}^{0}}^{2}$m)
Troposphere mapping function	GMF	GMF
Initial troposphere RZTD (variance)	0.0 m (0.15^2^ m^2^)	0.0 m (0.15^2^ m^2^)
Initial ambiguity (variance)	derived from pseudorange (30^2^ cycle^2^)	derived from pseudorange (30^2^ cycle^2^)
Initial slant ionosphere delay (variance)	0.0 m ($0.05^{2}*BL^{2}/100$m^2^)	0.0 m ($0.03^{2}*BL^{2}/100$m^2^)
Process noise of troposphere RZTD	$10^{-4}$m/$\sqrt{s}$	$10^{-4}$m/$\sqrt{s}$
Estimator	Kalman filter	Kalman filter

**Table 3:** Average performance improvement rate (%) of the 15 baselines in terms of TTFF, fixed rate, STD, and RMS error on DOY016, 092, 190, and 289 when comparing the GPT2-constrained model with the traditional model.

Improvement Rate (%)	BDS				GPS				GPS/BDS			
	016	092	190	289	016	092	190	289	016	092	190	289
TTFF	45.2	26.7	27.9	23.9	35.4	26.8	32.7	29.5	38.2	31.7	32.8	29.2
Fixed rate	7.5	4.8	10.3	7.5	4.1	2.9	2.3	3.2	0.8	0.5	0.4	1.1
STD	54.0	50.3	47.7	57.7	24.8	28.1	28.4	24.6	41.3	34.7	24.0	36.0
RMS error	44.2	39.5	36.3	40.2	22.9	17.3	15.7	17.8	33.1	14.7	12.1	19.3
